# Galactofuranose-Related Enzymes: Challenges and Hopes

**DOI:** 10.3390/ijms21103465

**Published:** 2020-05-14

**Authors:** Mateja Seničar, Pierre Lafite, Svetlana V. Eliseeva, Stéphane Petoud, Ludovic Landemarre, Richard Daniellou

**Affiliations:** 1 Institut de Chimie Organique et Analytique, CNRS UMR 7311, Université d’Orléans, Rue de Chartres, BP 6759, CEDEX 2, 45067 Orléans, France; mateja.senicar@univ-orleans.fr (M.S.); pierre.lafite@univ-orleans.fr (P.L.); 2 Centre de Biophysique Moléculaire, CNRS UPR 4301, Rue Charles Sadron CS 8005, 45071 Orléans, France; svetlana.eliseeva@cnrs.fr (S.V.E.); stephane.petoud@cnrs-orleans.fr (S.P.); 3 GLYcoDiag, 45200 Chevilly, France; landemarre@glycodiag.com

**Keywords:** glycobiology, galactofuranosyl transferase, galactofuranoside hydrolase, pathogen diagnostics, carbohydrate-based therapeutics

## Abstract

Galactofuranose is a rare form of the well-known galactose sugar, and its occurrence in numerous pathogenic micro-organisms makes the enzymes responsible for its biosynthesis interesting targets. Herein, we review the role of these carbohydrate-related proteins with a special emphasis on the galactofuranosidases we recently characterized as an efficient recombinant biocatalyst.

## 1. Introduction

**Figure 1:**
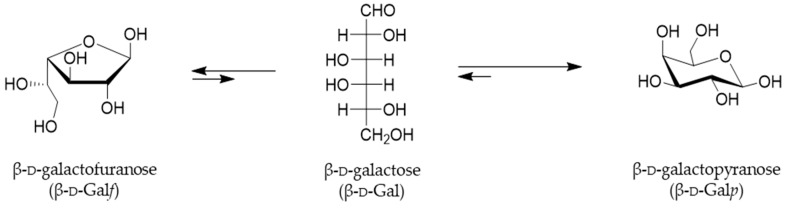
Representation of the equilibria between cyclic forms of d-galactose in solution. The rapid interconversion of cyclic Gal*f* and Gal*p* takes place through an acyclic form, and the favorable equilibrium for Gal*p* are indicated by the arrows [3].

While the existence of less common l-galactose was only reported in some plants, algae and snail glycans, d-Galactose is a hexoaldose, C-4 epimer of glucose, widely distributed in nature and it can be found in both pyranose and furanose configurations [1]. Indeed, in both solid and solution states, hexoses exist in a cyclic hemiacetal form and occupy either the most sterically and thermodynamically favored six-membered pyranosyl or five-membered furanosyl forms. Both cyclic forms of galactose, Gal*f* and Gal*p*, are interconverted through a linear form, which is present only in an instantaneous extent (Figure 1) [2].

Nevertheless, galactofuranose is by far the most widespread hexofuranose in nature and its occurrence was recently reviewed [4]. This thermodynamically disfavored hexofuranose is absent in mammalian glycoconjugates, but is present in living organisms ranging from archaea and bacteria, to protozoa, fungi and plants where it forms glyosidic linkages mainly in β-anomeric configuration, although rare examples in which an α-anomeric configuration occurrence can also be found [5]. In mammals, only the galactopyranose is found as a ubiquitous form. Exceptionally, it can also be present as free monosaccharide in nature where it is commonly linked to other molecules like glucose, constituting simple disaccharide structures (lactose or milk sugar), or as a constituent of glycans in complex glycoproteins (ABO blood group antigens) and glycolipids [1,6].

The interest for furanoses dates back to the beginning of the 20th century, and the beginning of the carbohydrate chemistry occurred when the first synthetic methods for the selective synthesis of glycofuranosides were established [7]. Galactofuranose was first identified in 1937 as a component of a fungal extracellular polysaccharide, galactocarolose, produced by *Penicillium charlesii* [8,9]. Only years later, the synthesis of the first β-d-galactofuranosides was reported [10]. Over the decades, galactofuranose has been found in many naturally occurring molecules originating from a variety of organisms, not necessarily pathogenic. Lately, the interest for this unusual carbohydrate has not decreased due to findings of its xenobiotic and immunogenic properties [4]. In addition, its occurrence in pathogenic organisms make the enzymes related to its biosynthesis of outmost interest for the glycoscientists at large, as these proteins, if cloned, overexpressed and well-characterized, can be further used as innovative biocatalysts, therapeutic targets or for diagnosis. All these aspects will be discussed herein.

## 2. Occurrence in Nature

In the following sections, the occurrences of β-d-Gal*f* units will be presented. Here, the intention is not to be exhaustive and to provide a complete overview of all natural Gal*f*-containing structures, but rather to focus on major structures, particularly found in prokaryotes and in two different classes of eukaryotes, fungi and protozoa and to illustrate them with the representative and most studied examples. Thereby, the focus will mainly be on pathogenic organisms such as bacteria *Mycobacterium tuberculosis*, fungus *Aspergillus fumigatus* and protozoa *Leishmania major*.

### 2.1. In Bacteria

In both Gram-positive and Gram-negative bacteria, Gal*f* is either part of a homopolymer formed with a Gal*f* disaccharide as the repeating unit, or part of a heteropolymer, linked to another monosaccharide, frequently galactopyranose, forming regular glycans that are sometimes branched [5]. These glycans, composed of Gal*f* units, are usually part of complex glycoconjugates structures that are constituents of bacterial cell walls. Some of these bacteria are highly pathogenic, and the presence of Gal*f*-containing conjugates appears to be an essential parameter related to their virulence. These conjugate include lipopolysaccharide (LPS) O-antigens of *Escherichia coli* K-12 and enteroinvasive *E. coli* O164 as well as the galactan-I repeating unit of O-antigen from *Klebsiella pneumoniae* [4,6].

**Figure 2:**
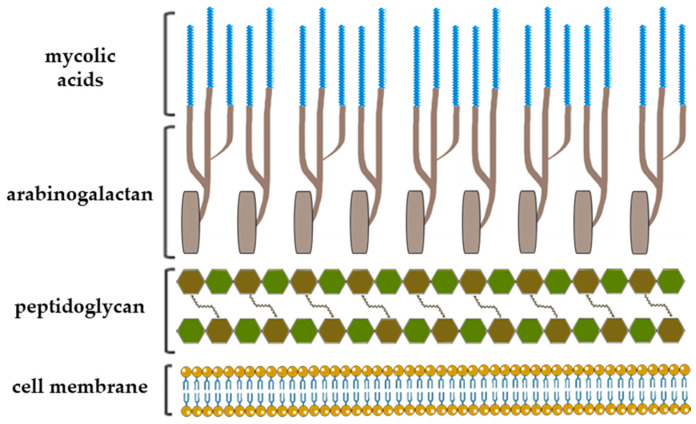
A schematic representation of the *Mycobacterium tuberculosis* cell wall structure with depiction of the three major cell wall features, including peptidoglycan, arabinogalactan and mycolic acids [13,14].

The notable example of highly complex structure constituted largely of rare carbohydrates and lipids is the mycobacterial cell envelope where Gal*f* has a crucial constructive role [11]. After several decades of successful chemotherapeutic treatments and vaccination preventions, *Mycobacterium tuberculosis* has emerged as being multidrug resistant, and tuberculosis became one of the major causes of mortality worldwide [4,12]. The cell envelope of *Mycobacterium tuberculosis* is a very thick, hydrophobic structure possessing very limited permeability. It consists of three main structural components: typical prokaryotic plasma membrane, cell wall and outer membrane, also referred as mycomembrane [13]. The entire complex of cell wall is noncovalently attached to plasma membrane with the bottom peptidoglycan layer, which, via a disaccharide linker, connects to the central glycan core, highly branched arabinogalactan polysaccharide. In addition, two-thirds of the arabinans are terminated by a cluster of mycolic acids that form outer membrane layer and capsular segment containing a variety of loosely attached proteins, lipids and polysaccharides (Figure 2) [6,13].

Arabinogalactan is a cell wall core assembly composed of arabinofuranose and galactofuranose that constitutes two distinct structural motifs: arabinan and galactan. The galactan is a linear polymer and consists of approximately 30 altering β-d-Gal*f*-(1→5) and β-d-Gal*f*-(1→6) residues that bear highly branched arabinan chains, the inner backbone of which contains about 35 (1→5)-α-d-Ara*f* residues [6,12,13].

The mycobacterial cell wall is essential for its viability and is largely responsible for the ability of the macrophage to survive and escape the hostile environment. It is evident that β-d-Gal*f* is an anchoring platform for the arabinogalactan complex; therefore, it remains a central focus as a drug target [12,15].

On the other hand, the motif β-d-Gal*f*-(1→6)-α-d-*Galf* was identified as part of exopolysaccharides (EPS) of non-pathogenic strains such is *Lactobacillus rhamnosus*, isolated from human intestinal flora, [4] *Bifidobacterium catenulatum* [16] or *Streptococcus thermophilus*, produced in skimmed milk [17]. It is also interesting to note that the sulfated polysaccharide-peptidoglycan complex, produced by soil bacteria *Arthrobacter* sp., contains a repeating trisaccharidic motif and shows antitumor activity [4,18].

Although α-d-Gal*f* is rare, it can be found incorporated as a single, internally or terminally positioned unit in the glycan core motifs [5]. It is the case in the anaerobic eubacteria *Clostridium thermocellum* and *Bacteroides cellulosolvens*, which produce a high molecular-mass cellulose binding protein complex, the cellulosome. The latter is glycosylated and within tetrasaccharide units, an internal α-d-Gal*f*(1→2) is linked to Gal*p* [5].

### 2.2. In Fungi

**Figure 3:**
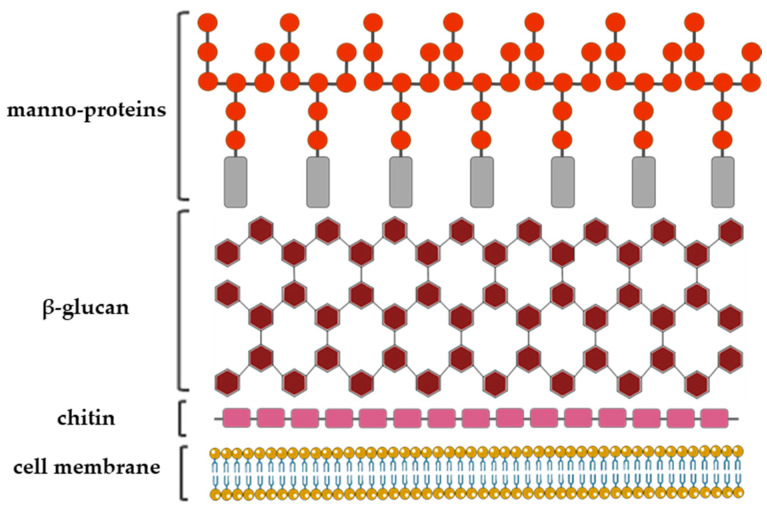
A schematic representation of the *Aspergillus fumigatus* cell wall structure with a depiction of the three major cell wall features, including chitin, glucan and galactomannan **[14,23]**.

The fungal cell wall structural organization and composition is generally layered. It consists of the inner, relatively conserved chitin core followed by branched glucan, which is bound to the outer, more heterogeneous layer composed of proteins and/or other polysaccharides the composition of which depends on the fungal species (Figure 3) [19].

In numerous pathogenic fungi species, for examples: *Penicillium*, *Histoplasma*, *Cryptococcus* and *Aspergillu*s, Gal*f* has been found as a part of the cell wall glycan [20].

*Aspergillus fumigatus* causes aspergillosis, one of the main fungal infections in humans, especially in immunodeficient patients. Interestingly, a single Gal*f* is essential for the immunoreactivity of the glycan and therefore they have been studied as in vitro markers for the early diagnosis of the invasive aspergillosis [5]. It is remarkable since 5% of the dry weight of *A. fumigatus* consists of Gal*f*, and as it is a quite abundant monosaccharide found and described at least in four different molecules, i.e., galactomannan and its secreted form, glycoproteins, and within glycosphingolipids [3]. Galactomannan is a main exopolysaccharide structural form of the cell wall, composed of (1→2)- and (1→6)-α-d-mannopyranoside core chain with around four to ten units of (1→5)-β-d-Gal*f* residues. This galactomannan is also secreted and can be found as a free, soluble polysaccharide in the medium. In addition, β-Gal*f*-(1→5)-β-Gal*f* was identified in both *O*- and *N*-linked glycans from the peptidogalactomannan and glycoinositolphosphoceramide [21,22].

Other, noteworthy examples of d-Gal*f*-containing molecules present in other fungal species, include the less common α-d-Gal*f* found together with β-d-Gal*f* in varianose, extracellular polysaccharide produced by *Penicillium varians* [24]. The same repeating units of varianose have been found in the cell wall of exopolysaccharides of *P. vermiculatum* and *Talaromyces flavus* while *Apodus deciduus* contains the (1→2)-Gal*f* disaccharide only in α-configuration [5].

### 2.3. In Protozoa

The presence of Gal*f* in eukaryotic protozoa was reported in the early 1980s in the family of *Trypanosomatidae*, which includes for humans and animals parasitic genera *Trypanosoma* and *Leishmania*. The glycoconjugates in these parasites have been characterized in detail and to our knowledge, no other examples of protozoa except trypanosomatids *Crithidia* spp. and *Endotrypanum schaudinni* containing Gal*f* have been reported [25,26].

Protozoan parasites of the genus *Leishmania* have two different life cycle stages, the flagellate promastigote stage in insects and the amastigote stage in mammalian phagocytic cells, during which many morphological and structural changes of the glycoclayx membrane occur. The related Gal*f* glycosylated membrane macromolecules are well studied in *Leishmania major*, a causative agent of leishmaniosis, and include two types of molecules, lipophosphoglycans (LPGs) and glycoinositolphospholipids (GIPLs). LPG is a major glycoconjugate that covers entire surface of promastigote and contains an internal hexasaccharide β-Gal*f*(1→3)-Man*p* core, which is conserved among all *Leishmania* species. The GIPLs are present at the membrane surface at a ten times higher amount than LPGs, and contain the same repeating unit, only externally positioned. Both glycolipid complexes, LPG and GIPL, are involved in the virulence and survival of the parasite, and their unconventional hexasaccharide core has become a target for the search for new drugs [27,28]. Similar LPG and GIPL are present in *Trypanasoma cruzi*, the causative agent of the Chagas disease [4].

### 2.4. In Other Organisms

**Figure 4:**
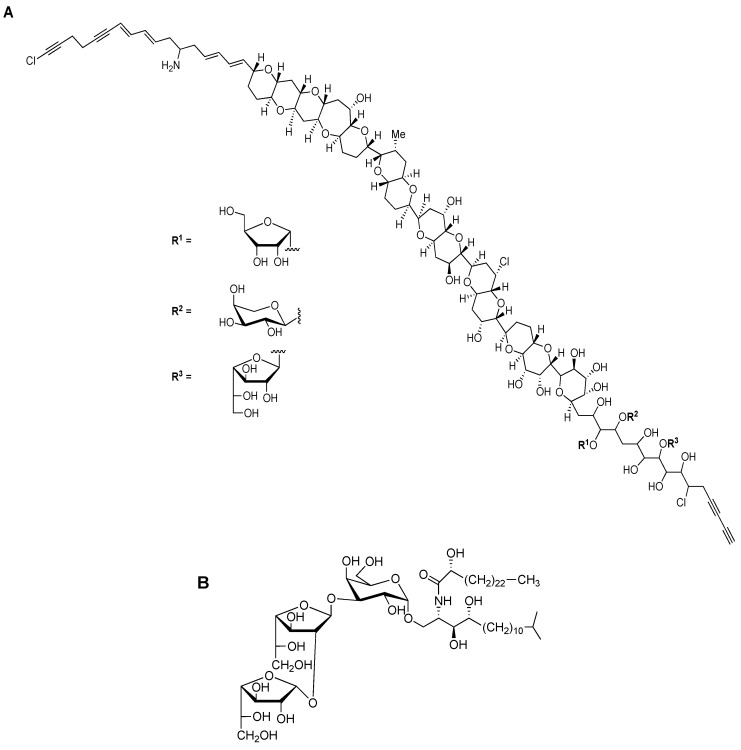
Chemical structures of Gal*f*-containing glycoconjugates. (**A**) The structure of toxin prymnesin-1, produced by *Prymnesiou parvum*. (**B**) The structure of agelagalastatin, glycosphingolipid produced by *Agelas* sp. [4].

Beside previously mentioned groups of organisms, other eukaryotic organisms expressing different Gal*f*-containing surface molecules include certain plants, lichens and marine organisms. Even if rare in terrestrial plants, Gal*f* was first described in 1981 as a part of the cell wall glycoproteins in the unicellular green algae *Chlamydomonas reinhardii* [29]. Molecularly unique, prymnesin-1 toxin, isolated from red tide alga *Prymnesiou parvum*, contains β-d-Gal*f* residue linked to C_90_ unbranched carbon chain (Figure 4A) [4,30].

Among marine organisms, especially sponges of the genus *Agelas* sp. glycosphingolipid agelagalastatin was described as containing the trisaccharide α-Gal*f*(1→2)-β-Gal*f*-(1→3)-Gal*p*, and it showed an antitumoral activity (Figure 4B) [31]. Steroidal glycosides and gangliosides containing Gal*f* were isolated from the starfish *Anthenea chinensis* and *Achanthaster planci* respectively [4,5]. The presence of Gal*f* was also reported in the nematode *Caenorhabditis elegans*, one of the model organisms in molecular and animal biology studies, but no reports about the presence in the mammals, including humans, have been reported so far [32].

## 3. Aspects of Enzymatic Biosynthesis and Metabolism

**Figure 5:**
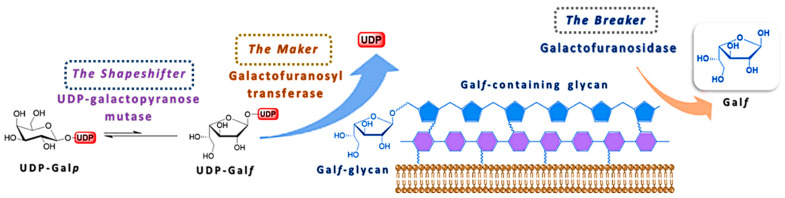
Schematic representation of the Gal*f*-glycan biosynthesis and the involvement of the three main enzymes, UDP-galactopyranose mutase (UGM), galactofuranosyltransferase (Gal*f*T) and galactofuranosidase (Gal*f*-ase).

The uniqueness of Gal*f* as a central component of cell surface glycoconjugates of human pathogens has led to an increased interest for the elaboration of its biosynthesis. The detailed mechanism of its biosynthesis and metabolism has been difficult to elucidate due to the instability of Gal*f* itself and to the fact that only few of these enzymes have been isolated and studied in the purified form. From the studies available so far, it is evident that two putative enzymes, UDP-galactopyranose mutase (UGM) and galactofuranosyltransferase (Gal*f*T) catalyze two synthetic steps and give a rise to a galactofuran extracellular conjugates, while the exact function of galactofuranosidase (Gal*f*-ase), as the degrading enzyme, and its overall contribution to the Gal*f* metabolism remains unclear (Figure 5) [3,6]. In addition to the confirmation of the canonical metabolism scheme and of the absence of alternative pathways, the identification of the three classes of enzymes involved and the coding genes, the efforts to understand their in vivo interaction and importance for viability or virulence, possibly in one organism, are still ongoing.

### 3.1. UDP-Galactopyranose Mutase (UGM)

**Figure 6:**
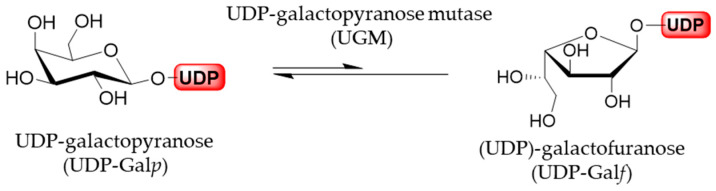
UGM-catalyzed izomerization of UDP-Gal*p* and UDP-Gal*f*.

Generally, furanoses, as nucleotide activated monomer donors, are transferred to the acceptors, growing oligomers or polymers, by glycofuranosyltransferases. In vivo, these furanose donors are generated from the corresponding pyranoses by the activity of pyranose mutases. The sole source of all Gal*f* residues is uracil diphosphate (UDP)-galactofuranose (UDP-Gal*f*), which originates directly from the UDP-galactopyranose (UDP-Gal*p*). In addition, UDP-Gal*p* is biosynthesized in all species from glucose, originating from a de novo synthesis pathway, or in some organisms it may be formed from free galactose by galactose salvage pathway. The activated UDP-Gal*p* is interconverted, through reversible ring-contraction, into UDP-Gal*f* by the cytosolic enzyme UDP-Gal*p* mutase. The equilibrium of the UGM-catalyzed reaction greatly favors thermodynamically more stable UDP-Gal*p* by the 11:1 ratio (Figure 6) [1,33].

It was established first in 1971 in *Salmonella typhimirium* that UDP-Gal*p* is precursor of UDP-Gal*f*. More than twenty years later, in 1996, the *glf* gene encoding for a UGM was first identified and cloned from *E. coli* [34]. Soon after the existence of homologue genes and UGMs was confirmed and characterized in several other prokaryotes, bacteria *Klebsiella pneumoniae*, *Mycobacterium tuberculosis* and *Campylobacter jejuni*, as well as in protozoan eukaryotes *Leishmania major* and *Trypanosoma cruzi*, fungus *Aspergillus fumigatus* and nematode *Caenorhabditis elegans* [1,35].

In 2001, the first crystal structure of UGM from *E. coli* was reported [35]. Currently, a total of 58 crystal structures of UGM obtained from nine organisms have been deposited in the Protein Data Bank (PDB). Some of these structures have been crystalized in both, the active (reduced) and the inactive (oxidized) states, as well as complexed with different substrates or ligands. The structures gave insight into its tertiary structure and revealed that the overall architecture of active site consists of conserved amino acid residues and that generally, UGMs are flavoenzymes working by the unique mechanism involving flavine adenine dinucleotide (FAD) cofactor in its reduced form [35,36].

Since the UGM is at the center of Gal*f* biosynthesis, the mutagenesis or the deletion of its genes is enabling to study the impact of UGM absence and, consequently the Gal*f* absence, on the in vivo organism integrity.

### 3.2. Galactofuranosyltransferase (GalfT)

**Figure 7:**
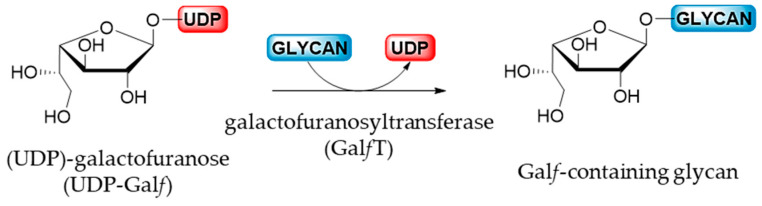
Biosynthesis of Gal*f*-glycan catalyzed by Gal*f*T.

After the isomerization catalyzed by UGM, the newly produced UDP-Gal*f* is transported, via the UDP-Gal*f* transporter only in eukaryotes, from the cytosol into the Golgi apparatus where the glycosylation by galactofuranosyltransferases takes place. Gal*f*Ts are the final enzymes involved in the biosynthesis of Gal*f-*containing molecules by a catalyzed reaction that corresponds to the nucleophilic substitution of an acceptor on the anomeric position of an activated sugar donor (Figure 7) [6]. Probably due to the high costs and difficulties in obtaining the activated donor combined with the limited access of the relevant enzymes, Gal*f*Ts were less studied than the mutases [5]. However, at the moment, only a few Gal*f*Ts of prokaryotic, exclusively bacterial, and eukaryotic origin have been cloned and characterized [1,5,6].

By far the most studied prokaryotic Gal*f*Ts are Gl*f*T1 and Gl*f*T2, two essential transferases for the biosynthesis of *Mycobacterium tuberculosis* galactan. Interestingly, they share a low sequence homology and are coded by different genes; Gl*f*T1 is encoded by the *Rv3782* gene and GlfT2 by the *Rv3808c* gene, respectively. Both transferases have been expressed as recombinant enzymes and characterized. The subsequent studies elucidated role of each transferase and demonstrated that Gl*f*T1 is responsible for the transfer of the two first Gal*f* on the acceptor substrate, whereas Gl*f*T2 pursues the polymerization of the resulting acceptor by introducing approximately 30 remaining monosaccharides [37,38,39].

Since both use UDP-α-d-galactofuranose as the donor and the change from α-stereochemistry in UDP-Gal*f* to the β-stereochemistry of the newly synthesized glycan, indicate that catalysis follows an inverting mechanism. They are also characterized as being bifunctional because they are synthetizing both β-Gal*f*-(1→5)-Gal*f* and β-Gal*f*-(1→6)-Gal*f* linkages between Gal*f* residues. Gl*f*T2 has been more extensively studied than Gl*f*T1; therefore, it is the only Gal*f*T with reported crystal structure and the confirmed presence of only one catalytic site [38,39].

Few other well-characterized prokaryotic Gal*f*Ts include WbbI from *Escherichia coli* K-12 that is able to transfer β-(1→6)-Gal*f* to α-glucose [40] and WbbO from *Klebsiella pneumoniae*, another bifunctional transferase that couples β-Gal*f*-(1→6)-Gal*p* [41].

Until recently, LPG1 from *Leishmania major* was the first and only described eukaryotic Gal*f*T. In an extensive study from 2018, all the four putative transferases (LPG1, LPG1G, LPG1L and LPG1R) encoded in the *L. major* genome were cloned, overexpressed and their kinetic parameters determined. It was demonstrated that they are able to use both UDP-Gal*f* and UDP-Gal*p* as donor substrates [42].

Another two-known eukaryotic Gal*f*Ts are of fungal origin, GfsA from *Aspergillus nidulans* and A*f*gfsA from *A. fumigatus*. As it was shown, both enzymes are localized in the Golgi apparatus and use UDP-Gal*f* as a sugar donor [43,44]. Moreover the galactofuranosyltransferase activity was demonstrated in *P. fellatum* through the incorporation of radiolabelled Gal*f* into peptidophosphogalactomannan prepared from its membrane, but no further investigations were conducted [45].

### 3.3. Galactofuranosidases

Although the metabolism of β-d-galactofuranosides has been extensively studied, mostly in the infectious microorganisms such as *Mycobacteria*, *Trypanosoma*, *Leishmania* and *Aspergillus*, there are only few reports related to these enzymes, especially galactofuranosidase Gal*f*-ase.

Over the past forty years, Gal*f*-ase has been identified as being responsible for the degradation of the d-Gal*f* containing glycoconjugates [5,6]. In 1977, a specific extracellular exo-β-d-galactofuranosidase (exo-β-d-Gal*f*-ase) was the first one isolated and partially purified from *Penicillium fellutanum* (ex type of *Penicillium charlesii*) culture filtrates. The enzyme catalyzed the hydrolysis of β-galactofuranosides only and was incapable of cleaving α-L-arabinofuranosides, the pentosyl homologs. The enzyme was stable to freezing and thawing and reached optimum activity between pH 3 and 4 at the temperature of 47 °C [46].

Later, extracellular exo-β-d-Gal*f*-ases were described and/or purified from the culture medium of filamentous fungi such as *Helminthosporium sacchari* [47,48], *Trichoderma harzianum* [49], *Penicillium* and *Aspergillus* species [50], among which the one from *P. fellutanum* have been the most studied [46,51,52,53], *Aspergillus niger* [54] and protozoa *Trypanosoma cruzi* [55]. All the known β-Gal*f*-ases were exclusively exo enzymes and mostly have been of fungal origin.

There are only two reported endo-β-d-Galf-ases, isolated from fungus *Penicillium oxalicum* [56] and from bacteria *Bacillus* sp. [57]. The first endo-β-d-Galf-ase, purified in 1992 from supernatants of *P. oxalicum* autolysed cultures, hydrolyzed specifically β-d-(1→5)-linked galactofuranose residues. This was the first endo-β-d-Galf-ase for which optimum pH, stability properties, substrate specificity and kinetic characteristics were determined [56].

Only a few specific β-d-Gal*f*-ases have been reported and mostly detected in fungal species as an extracellular enzyme, which is secreted into the medium in low quantities.

In the absence of the identified Gal*f*-ase gene and overexpressed as a recombinant protein, their production was challenging. In order to induce a higher level of enzyme secretion, the synthetic growth medium was supplemented with various monosaccharides, disaccharides or polysaccharides [51], sometimes with those containing galactofuranoside residues [54,57] or the media from natural sources, like apple juice [50] was used. The production of Gal*f*-ase in *A. niger* were induced to significant levels in the presence of Gal*f* containing glycoconjugates (mycelial wall extracts) [54] and in *P. fellutanum* and *A. fumigatus* when the medium was depleted of glucose [46,50,51,58] or when the glucose was replaced by galactose as the carbon source [59].

Mostly, Gal*f*-ases were purified from *P. fellutanum* culture filtrates and have become model enzymes in characterization studies [46,47], but Gal*f*-ase were also commercially available from crude enzyme preparations [49,60] or from *T. cruzi* cell lysates [55].

To detect low levels of Gal*f*-ase in culture media, to isolate it and to possibly purify it, an array of Gal*f*-containing glycoconjugates, acting like potential substrates or inhibitors, was synthetized and assayed. Several conjugates proved to be good inhibitors, such as alkyl, aryl and heteroaryl 1-thio-β-d-galactofuranosides [59,61]. An affinity chromatography system was also developed using two inhibitors, 4-aminophenyl thio-β-d-galactofuranoside and d-galactono-1,4-lactone, as the immobilized and eluting ligands [52].

These early studies presented the pioneering work in the Gal*f*-ase research. In most cases, properties were determined using partially purified or even crude enzyme preparations and one of the main problems was the lack of a simple and sensitive quantitative method for the detection of their catalytic activity as well as a standardized substrate. The activity was usually determined by measuring the released galactose during the hydrolysis of galactofuranose-containing exopolysaccharide preparations of natural origin or methyl β-d-Gal*f* by the galactose oxidase method. Already experimentally established, the use of nitrophenyl glycosides, widely used as substrates for estimating the activity, kinetics and specificity of glycosidases, was extended to the *para*-nitrophenyl β-d-galactofuranose (*p*NP-β-d-Gal*f*) [50,62], which became a standardized and commercially available [54] substrate for assaying galactofuranosidases.

A colorimetric assay with *p*NP-β-d-Gal*f* as a substrate was extensively used. However, this substrate was not recognized by the endo-β-d-Gal*f*-ase from *Bacillus* sp. [57] and exo-β-d-Gal*f*-ase from *T. cruzi* [55] and could be attributed to a particular substrate specificity, relating to aglycone.

The diversity of organisms, experimental conditions and substrates were employed during these research studies making it very difficult to establish a unanimous and general conclusion. However, they showed reliable evidence that suggested some common characteristics, which are outlined (Table 1). The substrate specificity in respect to the glycon moiety from either natural or artificial substrates was exclusively towards β-d-Gal*f*. The enzymes proved to be stable over a longer period of time, even as crude preparations, and showed optimal activity in acidic conditions that are usual for the enzymes of invertebrates, (pH 4–5), and the stability at the temperatures up to 40 °C.

**Figure 8:**
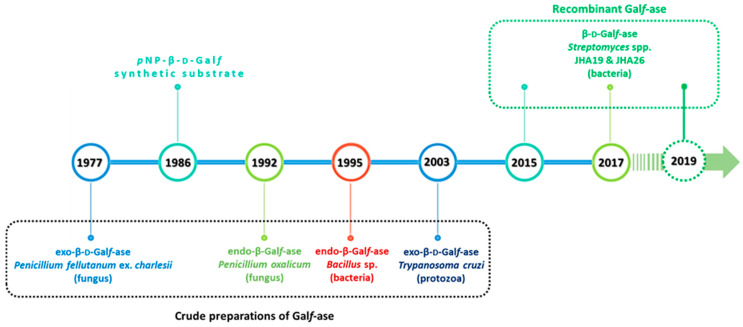
Schematic representation of the timeline of Gal*f*-ase discoveries and studies over the past forty years.

Although the first Gal*f*-ase was described back in 1977, and several exo- and endo-Gal*f*-ases were purified from the culture supernatants and cell lysates of filamentous fungi, bacteria and protozoa, the genes encoding these enzymes were not identified and expressed earlier, nor their amino acid sequence determined (Figure 8).

Recently, in 2015, based on the draft genome sequence analysis of soil, Gram-positive bacteria *Streptomyces* sp., strain JHA19, an open reading frame that encoded Gal*f* specific enzyme was identified. Based on the sequencing results, the genome size was 7.7 Mb and the entire Gal*f*-ase open reading frame (ORF) fragment contained 2361 base pairs (bp), which encoded 786 amino acids [63].

The Gal*f*-ase gene fragment was cloned, expressed and purified as a recombinant Nus and double 6xHis-tagged fusion protein. The enzyme was described as an exo-type Gal*f*-ase that hydrolyzed galactomannan, the naturally occurring Gal*f*-containing oligosaccharide, extracted from *A. fumigatus* cell wall, as well as the artificial substrate *p*NP-β-d-Gal*f*. No activity was observed with other *p*NP furanosyl and pyranosyl glycoconjugates, including *p*NP-α-L-Ara*f*. The optimal pH was found to be 5.5, and the enzyme was stable at temperatures up to 40 °C with the *K*_M_ value of 4.4 mM (Table 2). This was the first report of an identification and cloning of a gene coding for the Gal*f*-specific Gal*f*-ase enzyme that does not also exhibit arabinofuranosidase (Ara*f*-ase) activity [64].

Another Gal*f*-ase gene was also found later in the genome of *Streptomyces* sp., strain JHA26, coding for 869 amino acids. The Gal*f*-ase specific enzyme was expressed, purified and characterized with the highest activity at pH 4.5 and temperature stability up to 45 °C and with *K*_M_ of 6.8 mM for *p*NP-β-d-Gal*f* as a substrate [65,66].

The most recent addition of cloned enzymes is the one from 2019, which provided the complete biochemical and kinetic characterization of subcloned *Streptomyces* spp. JHA16 recombinant Gal*f*-ase to date. This *N*-terminal 6xHis-tagged Gal*f*-ase proved to be an efficient and stable biocatalyst exclusively towards the synthetic substrate *p*NP-β-d-Gal*f* possessing a *K*_M_ value of 0.25 mM and the highest activity at pH 4.5, temperature stability up to 60 °C as well as stability towards multiple freeze and thaw cycles as a crude preparation (Table 2) [67].

## 4. Galactofuranose Antigens—Therapeutic and Diagnostic Target

The search for molecules that are specific to pathogenic microorganisms, pathogen-associated molecular patterns (PAMPs), preferably surface-exposed, conserved in pathogens and absent in host organisms, to circumvent a potential risk of interference, has seemingly led to one potential target, the galactofuranose [68,69].

Biomolecules involving galactofuranose have attracted interest because they fulfill these requirements and their presence in many pathogens suggests to be an advantageous element of survival and is considered essential for their virulence [4]. Hence, galactofuranose is as a new and interesting candidate as a target in medical or biotechnological applications.

Possibilities to exploit the absence of this unusual monosaccharide in mammals arise from its biological significance in these pathogens. The impact of the galactofuranose deficiency on cell morphology and growth and its role in virulence was the focus of numerous research studies, predominately performed on eukaryotic pathogens of *Aspergillus* and *Leishmania* species. The modifications of the cell surfaces were most evident in fungi and resulted in aberrant morphological changes and growth reduction, leading to a hypersensitivity to drugs and osmotic stress. The lack of Gal*f* had a variable impact on the virulence capacity of *Leishmania*, *Trypanosoma* and *Aspergillus* species. While in *Leishmania major*, Gal*f*-deficient mutants presented only the initial delay in infection onset, in *L. mexicana* infectivity was not attenuated and in the related parasite *T. cruzi*, Gal*f*-containing strains were less infectious than those expressing Gal*p*. On the other hand *A. fumigatus* displayed attenuated virulence, which might be dose-related [3].

Interestingly, the virulence effects vary with the observed organisms, indicating that the Gal*f* involvement cannot be generalized and rather should be considered separately, related to specific pathogen species. Understanding the exact role and contribution of individual Gal*f*-containing glycoconjugates and Gal*f* itself on the morphology, survival and virulence, as well as its role in the immune response, remain to be clarified.

Therefore, the medical application of Gal*f*-based therapeutics or mimetics is still challenging and relies on the elucidation of the Gal*f* biosynthesis pathways.

However, the importance of Gal*f* as a diagnostic target showed it to be very useful. The presence of *Aspergillus* exoantigens of galactomannan (GM) origin, known to be secreted by the fungus during its growth in vitro and in vivo, is a specific indicator of this invasive disease and has become a detection target [70]. Monoclonal antibody detection methods for early serological diagnosis of galactomannan antigens, thus invasive pulmonary aspergillosis, have been experimentally developed since 1980s. It was not only until 1995 that a double-direct sandwich enzyme-linked immunosorbent assay (ELISA) was developed, that this assay employs a rat anti-GM monoclonal antibody, EB-A2, directed against the β-Gal*f*(1→3)-β-Gal*f* epitopes of GM. Today, two GM antigen detection kits are commercially available, the Pastorex *Aspergillus* and the Platelia^™^ *Aspergillus*. The Pastorex, latex agglutination test, has mostly been replaced by the Platelia^™^ *Aspergillus*, EIA, which has been available in Europe for more than 20 years and in the USA since 2003. To our knowledge, these are the only commercially available tests based on the detection of Gal*f* epitopes [71,72].

In parallel, a novel experimental procedure for the non-invasive detection of *Aspergillus* lung infection, based on antibody-guided positron emission tomography and magnetic resonance (immunoPET/MR) imaging, has been developed and tested. In 2016 the prototype version, [^64^Cu]DOTA-mJF5 tracer, showed that a mouse monoclonal antibody (mJF5) specifically binds to the mannoprotein antigen, pathogen related only, and that the antibody-labeled, radionucleotide ^64^Cu and DOTA chelator complex, allows for the combined PET imaging. This highly specific [^64^Cu]DOTA-mJF5 tracer allows repeated imaging and distinguishes aspergillosis from pulmonary inflammation and bacterial lung infections [73].

Only a year later, the same team reported the development of a humanized version of the JF5 antibody (hJF5). This new, [^64^Cu]NODAGA-hJF5 tracer showed not only improved imaging capabilities but also a high specificity towards Gal*f*(1→3)-β-Gal*f* epitopes, present in a mannoprotein antigen released by *Aspergillus* during lung infection. This was the first time that Gal*f*-specific, antibody-guided in vivo imaging has been used for non-invasive preclinical diagnosis of a fungal lung disease [74].

These recent experimental techniques based on Gal*f* specific antibodies are still under development and are greatly contributing towards more specific targeting of epitope patterns. The related imaging and diagnostic aspects have yet to be explored.

## 5. Conclusions

The impact of modern biotechnology and recombinant DNA technology has made enzymes available in an economically feasible approach. It enabled a whole new diversity of enzymes to be accessed in the field of glycoenzymes. In parallel carbohydrate-based materials have emerged in an increasing number of applications in the food, feed, pharmaceutical and other industries. To access these carbohydrate structures that are in high demand, natural glycoenzyme catalysts have provided over the last few decades an alternative to chemical synthesis. A current area of interest is broadening the search spectrum to rare glycan substrate specific glycoside hydrolases, preferably, from narrow group of organisms involved in pathological conditions, in order to create altered characteristics, various functions and application possibilities within protein engineering. The biocatalyzed synthesis of galactofuranosyl containing conjugates still represents an emerging area due to our limited knowledge about the interaction of the protein with this specific and rare carbohydrate. Recent findings in the field described in this review render it very promising.

## Figures and Tables

**Table 1:** Comparative properties of β-d-galactofuranosidases.

Enzyme	Species	Substrate	pH	T (°C)	M (kDa)	K_M_ (mM) ^†^	Year	Reference
extracellular exo-β-d-Gal*f*-ase	*Penicillium charlesii* (fungus)	pPGM ^a^	4	47	–	–	1977	[46]
β-Gal*f*-ase	*Helminthosporium sacchari* (fungus)	1-*O*-methyl-β-Gal*f*	4.2 & 5.2	38	–	–	1983	[47]
β-d-Gal*f*-ase	*H. sacchari* (fungus)	HS toxin ^b^	4.6	37	–	–	1983	[48]
extracellular β-d-Gal*f*-ase	*Penicillium* spp.	*p*NP-β-d-Gal*f*	5	30	–	–	1989	[50]
	*Aspergillus* spp. (fungi)							
endo-β-Gal*f*-ase	*Penicillium oxalicum* (fungus)	β-(1→5)-galactofuran	5	37	77	–	1992	[56]
exo-β-d-Gal*f*-ase	*Trichoderma harzianum* (fungus)	EPS ^c^	4–4.5	35–40	35	–	1992	[49]
endo-β-Gal*f*-ase	*Bacillus* sp. (bacteria)	__	6	37	67	–	1995	[57]
exo-β-d-Gal*f*-ase	*Penicillium fellutanum* (fungus)	*p*NP-β-d-Gal*f*	3–6	37	70	0.3	1999	[52]
extracellular β-Gal*f*-ase	*Aspergillus niger* (fungus)	*p*NP-β-d-Gal*f*	3–4	37	90	4	2001	[54]
exo-β-d-Gal*f*-ase	*P. fellutanum* (fungus)	1-*O*-methyl-β-Gal*f*	4–4.5	40	70	2.6	2001	[53]
exo-β-d-Gal*f*-ase	*Trypanosoma cruzi* (protozoa)	LPPG ^d^	–	–	55	–	2003	[55]

^†^ K_M_ values determined only for *p*NP-β-d-Gal*f* and its derivatives as a substrate. ^a^ pPGM—peptidophosphogalactomannan from *Penicillium fellutanum* ^b^ HS toxin—host-selective toxin from *Helminthosporium sacchari* ^c^ EPS—extracellular polysaccharides from *Penicillium digitatum* ^d^ LPPG—lipopeptidophosphoglycan from *Trypanosoma cruzi*.

**Table 2:** Comparative properties of recombinant β-d-galactofuranosidases.

Gal*f*-Ase	Substrate	pH	T (°C)	*K*_M_ (mM)	Year	Reference
*Streptomyces* sp. (*JHA19*)	*p*NP-β-d-Gal*f*	5.5	50	4.4	2015	[64]
*Streptomyces* sp. (*JHA26*)	*p*NP-β-d-Gal*f*	4.5	45	6.8	2017	[65]
*Galf-ase* (*JHA19*)	*p*NP-β-d-Gal*f*	4.5	60	0.25	2019	[67]

## Abbreviations

ELISA	enzyme-linked immunosorbent assay
EPS	exopolysaccharides
EPS	extracellular polysaccharides
FAD	flavine adenine dinucleotide
Gal*f*	galactofuranose
Gal*f*T	galactofuranosyltransferase
Gal*p*	galactopyranose
GIPLs	glycoinosinositolphospholipids
GM	galactomannan
HS toxin	host-selective toxin
LPGs	lipophosphoglycans
LPPG	lipopeptidophosphoglycan
LPS	lipopolysaccharide
ORF	open reading frame
PAMPs	pathogen-associated molecular patterns
PDB	Protein Data Bank
PET/MR	positron emission tomography/magnetic resonance
*p*NP-β-d-Gal*f*	*para*-nitrophenyl β-d-galactofuranose
pPGM	peptidophosphogalactomannan
UDP	uracil diphosphate
UDP-Gal*f*	uracil diphosphate galactofuranose
UGM	UDP-galactopyranose mutase
